# Rhamnocitrin Ameliorates the Intestinal Fibrosis in DSS-Induced Colitis Mice by Modulating Host-Metabolites and Remodeling the Gut Microbiome

**DOI:** 10.3390/antiox15050639

**Published:** 2026-05-18

**Authors:** Ming-Yu Zhang, Zhi-Zhu Ke, Pei-Lin Deng, Yi-Yan Qin, Shu-Lan Mo, Lin-Ting Qiu, Jie-Jing Xu, Chen-Xi Tong, Jia-Le Song

**Affiliations:** 1 Guangxi Key Laboratory of Environmental Exposureomics and Entire Lifecycle Health, Guilin Medical University, Guilin 541199, China; 18150022696@stu.glmc.edu.cn (M.-Y.Z.); kezhizhu@stu.glmc.edu.cn (Z.-Z.K.); Moshulan@stu.glmc.edu.cn (S.-L.M.); Xujiejing@stu.glmu.edu.cn (J.-J.X.); tongchenxi@stu.glmc.edu.cn (C.-X.T.); 2 Department of Nutrition and Food Hygiene, School of Public Health, Guilin Medical University, Guilin 541199, China; dengpeilin@stu.glmu.edu.cn (P.-L.D.); qinyiyan@stu.glmu.edu.cn (Y.-Y.Q.); qiulingting@stu.glmu.edu.cn (L.-T.Q.); 3 School of Laboratory Medicine and Biotechnology, Guilin Medical University, Guilin 541004, China; 4 Guangxi Colleges and Universities Key Laboratory of Medical Biotechnology and Translational Medicine, Guilin 541199, China

**Keywords:** rhamnocitrin, ulcerative colitis, gut–liver axis, gut microbiota, metabolomics

## Abstract

Ulcerative colitis (UC) is characterized by barrier disruption, microbiota dysbiosis, fibrosis, and impaired autophagy. We investigated the effects of Rhamnocitrin (Rha) in dextran sulfate sodium (DSS)-induced chronic UC mice using histological analysis, molecular assays, and multiomics profiling. Rha alleviated weight loss and colon shortening; improved mucus secretion and tight junction protein expression; suppressed NLRP3 inflammasome activation; activated autophagy via AMPK activation and consequent Akt/mTOR inhibition; and attenuated colonic fibrosis. Multiomics analysis integrating 16S rRNA sequencing, metagenomics, and metabolomics revealed that Rha remodels the gut microbiota and is associated with elevated levels of beneficial metabolites, including butyrate in the colon, glutamate and γ-aminobutyric acid in the liver, and α-linolenic acid in the serum. Correlation analysis revealed close associations between microbiota and metabolite alterations, and improved barrier integrity, reduced inflammation, and attenuated fibrosis. These findings suggest that Rha ameliorates chronic UC by modulating autophagy, microbiota composition, and host metabolism across the gut–liver axis.

## 1. Introduction

Ulcerative colitis (UC) is an idiopathic chronic inflammation of the bowel characterized by mucosal ulcerations, with the main clinical symptoms including watery diarrhea, abdominal pain, and hematochezia [1]. The prevalence rates of UC are increasing both globally and in developed nations, raising concern in terms of public health [2]. The underlying mechanisms in UC involve intricate interactions between genes, environmental factors, immune dysfunction, and the microbial flora in the bowel [3]. In turn, immune cell infiltration occurs in irritated bowel tissue, where these cells produce pro-inflammatory cytokines, leading to the apoptosis or shedding of cells in the bowel tissue. These events compromise the integrity of bowel tissues and increase permeability [4]. The activation of the NLRP3 inflammasome by PAMP or DAMPs leads to cleavage of Caspase-1 proteins, resulting in the secretion of IL-1β and IL-18, in turn potentiating bowel inflammation and hence, UC pathogenesis [5]. Overexpression of NLRP3 and its associated IL-1β has long been reported to disrupt intestinal integrity and lead to immune dysfunction in vivo and in human patients with UC [6,7,8].

The gut–liver axis was recognized very recently as an important factor in UC pathogenesis [9,10]. Impaired harmony of the intestinal microbiota and changes in the metabolic output of microbiota result in this condition. Commensal bacteria secrete short-chain fatty acids (SCFAs) that support colonocytes and reduce inflammation through GPR41 and GPR109A [11,12]. An impaired microbiota structure causes decreased secretion of SCFAs and increased secretion of harmful metabolites from conditionally pathogenic bacteria. An impaired barrier allows these harmful metabolites to enter the blood, triggering the NF-κB and NLRP3 inflammasomes, thereby exacerbating inflammation in the colon and triggering immune activation in the liver through the portal blood supply. These events give rise to vicious cycle interactions between the gut and liver, triggering both local and systemic inflammation [13,14]. Within this inflammatory context, the chief profibrotic cytokine TGF-β1 remains activated. TGF-β1 activates Smads to stimulate profibrotic fibroblast activation and overproduction of the ECM, thereby laying the basic nidus of inflammation-induced fibrosis [15]. The progression of inflammation and injury in the bowel further triggers connective tissue and sclerotic changes to induce bowel fibrosis [16].

Despite the focus of modern UC treatments on managing inflammation to achieve remission, approximately 30–50% of patients develop intestinal fibrosis during the decade following diagnosis [17]. This represents an irreversible pathological outcome resulting from long-term and recurrent inflammatory infiltration, characterized by intestinal tissue remodeling, thickening of the muscularis mucosa, shortening of the colon, dysfunction of colonic motility, and abnormal defecation [18,19]. This clinical challenge highlights the need to develop new strategies to regulate cellular homeostasis. Autophagy, a fundamental process for removing damaged organelles and maintaining cellular stability, represents a promising target. Our previous work demonstrated that impaired autophagy plays a key role in UC pathogenesis, and that activating autophagy with phytochemicals can alleviate intestinal inflammation [20]. Current clinical treatments for UC rely on drugs such as 5-aminosalicylic acid (5-ASA) and corticosteroids, but the efficacy of these treatments are limited, and they are not without risks of adverse effects (e.g., paradoxical abdominal pain and diarrhea, muscle pain, hypertension, and diabetes mellitus) [21,22]. Therefore, the exploration of natural and effective phytochemicals is clinically important for addressing this health problem. Rhamnocitrin (Rha), derived from *Nervilia fordii* (Hance) Schltr., exhibits a broad range of pharmacological activities against inflammation, tumors, oxidative damage, and fibrosis [23,24]. Our preliminary research has demonstrated the therapeutic potential of Rha in acute UC [25]. However, the mechanisms by which Rha alleviates the core symptoms of chronic colitis, restores barrier function, modulates autophagy, and suppresses fibrosis remain unclear. Consequently, the present study systematically assessed how Rha influences disease activity, inflammation, barrier integrity, autophagy, and fibrosis in chronic UC mice. In addition, we conducted integrated analyses of non-targeted metabolomics, fecal metagenomics, gut microbiota profiling, and SCFA levels to provide experimental evidence supporting the development of natural product-derived therapeutic and antifibrotic strategies for UC.

## 2. Materials and Methods

### 2.1. Drugs and Reagents

Rha (WKQ-0004393; purity ≥ 98%) was obtained from Weikeqi Biological Technology Co., Ltd. (Chengdu, China). DSS (9011-18-1) was provided by MP Biomedicals (Irvine, CA, USA). 5-ASA (R006214; purity ≥ 98%) was acquired from Yien Chemical Technology Co., Ltd. (Shanghai, China). Tween-80 (HY-Y1891), PEG300 (HY-Y0873) and DMSO (HY-Y0320C) were obtained from MedChemExpress (Monmouth Junction, NJ, USA). TRNzol reagent (DP424) and the FastKing DNA Dispelling RT SuperMix Plus kit (KR118) were obtained from Tiangen Biotech Co., Ltd. (Beijing, China). AB (G1560) and PAS (G1281) staining kits were obtained from Solarbio Life Science Co. (Beijing, China).

### 2.2. Animals and Chronic UC Model Establishment

Male C57BL/6 mice (SPF, 6 weeks old) were obtained from Hunan Silaike Jingda Laboratory Animal Co., Ltd. (Changsha, China, License No. SCXK 2024-0009). After 7 days of acclimation, 52 mice were randomly assigned to four groups: normal (NOR), chronic UC (DSS), 5-ASA (50 mg/kg), and Rha (50 mg/kg). In contrast to the normal group, the other three groups received 2% (*w*/*v*) DSS dissolved in drinking water for 28 days to establish a chronic colitis model. On days 14–28, the 5-ASA and Rha groups received the corresponding treatments by gavage (dissolved in 10% DMSO, 40% PEG300, 5% Tween 80, and 45% saline), whereas the other groups received the same volume of the vehicle solution. Body weight was recorded daily throughout the study. At the study endpoint, the mice were deeply anesthetized and then euthanized by intraperitoneal injection of 1.25% tribromoethanol (250 mg/kg). All animal studies were conducted under the authorization of Guilin Medical University’s Institutional Animal Care and Use Committee (GLMC-IACUC-202510124).

### 2.3. Disease Activity Index (DAI)

Body weight, fecal consistency, and stool bleeding were evaluated and documented every 2 days. DAI scores for each mouse were determined according to the criteria shown in Table S1, as previously described [26].

### 2.4. Histopathological Examination and Immunohistochemistry (IHC) Assay

Colon tissues were weighed, and their lengths were recorded upon collection. The colon tissue (1–2 cm) was fixed in 4% paraformaldehyde, while the remaining portion was rinsed with saline and stored at −80 °C. After dehydration and paraffin embedding, the fixed colon tissue sections were stained with AB, H&E, and PAS. IHC was used to quantify the expression of inflammation-related markers, TJ proteins, and fibrosis-related factors in accordance with our previous protocols [20,21].

### 2.5. Real-Time Quantitative PCR

Total RNA was isolated from colon tissues using TRIzol reagent (Invitrogen, Carlsbad, CA, USA), and cDNA was synthesized with a FastKing gDNA kit (Tiangen Biotech, Beijing, China). qPCR amplification was performed on a QuantStudio 6 Flex Real-Time PCR system (Applied Biosystems, Thermo Fisher Scientific, Waltham, MA, USA). All primer sequences are provided in Table S2. Relative transcript levels were quantified by the 2^−ΔΔCt^ approach, with *Gapdh* employed as the normalization reference [25].

### 2.6. Western Blot Analysis

Colon tissue samples (30 mg) were disrupted in 200 µL of RIPA buffer containing 1 mM PMSF. Following centrifugation, the supernatant was harvested, and the protein concentration was quantified by a BCA assay. Protein samples were separated by SDS-PAGE, electrophoretically transferred to PVDF membranes, blocked with 5% skim milk, and incubated with primary antibodies (Table S3) overnight at 4 °C, followed by incubation with HRP-conjugated secondary antibodies for one hour at 25 °C. Following three TBST washes, the bands were visualized with ECL and imaged using an SCG-W2000 chemiluminescence system (Servicebio, Wuhan, China) [27].

### 2.7. 16S rRNA Analysis

Genomic DNA was extracted from the fecal material using a CTAB-based method, and its quality and concentration were assessed by 1% agarose gel electrophoresis. The V3–V4 region of the bacterial 16S rRNA gene was amplified with the primers 341F and 806R on a thermal cycler (Bio-Rad T100, Bio-Rad Laboratories, Hercules, CA, USA). Amplification products were purified using Tiangen’s universal DNA kit (Tiangen Biotech, Beijing, China). The purified amplicons were subsequently analyzed on the Illumina NovaSeq platform (Illumina, San Diego, CA, USA) using a paired-end 2 × 250 bp strategy. Raw reads were analyzed through the QIIME2 workflow for quality control and feature table generation. Downstream analyses included calculations of alpha and beta diversity, profiling of taxonomic distributions at the phylum and genus levels, and differential microbial enrichment evaluation by LEfSe [28].

### 2.8. Detection of SCFAs

Fecal samples were ground in liquid nitrogen, extracted with 80% methanol, and centrifuged (12,000 rpm, 10 min) to remove proteins. After mixing with 150 μL of derivatization reagent, the resulting supernatant was incubated at 40 °C for 40 min. The mixture was subsequently diluted with 80% methanol. For SCFA quantification, 95 μL of the solution was mixed with 5 μL of the internal standard and injected into the LC-MS/MS system (AB Sciex Triple Quad 5500, SCIEX, Framingham, MA, USA) [29].

### 2.9. Metagenomic Sequencing and Analysis of Fecal Samples

Microbial DNA was isolated from 200 mg of fecal sample using the QIAamp PowerFecal Pro DNA Kit (Qiagen, Hilden, Germany). DNA concentration and quality were assessed using a NanoDrop spectrophotometer and a Qubit fluorometer (Thermo, Waltham, MA, USA). Qualified samples were subjected to library construction and Illumina sequencing. Following adapter ligation and indexing, paired-end 150 bp (PE150) sequencing was performed, with the quality of the raw reads first assessed using FastQC (v0.12.1). Quality control was subsequently performed using Trimmomatic (v0.39), which included the removal of adapter sequences and low-quality reads (parameter set: SLIDINGWINDOW:4:20 MINLEN:50) [30]. To eliminate potential contamination from host (mouse) genomic sequences, the quality-controlled reads were mapped against the mouse reference (GRCm39) by Bowtie2 (v2.3.5.1) [31], and all matching reads were removed. All subsequent analyses were conducted on the basis of the resulting clean, host-filtered metagenomic data. Species composition analysis was performed using MetaPhlAn 4 (metagenomic phylogenetic analysis) [32] to obtain microbial community profiles from the phylum to the species level. Concurrently, the high-quality reads were processed through the HUMAnN (v3.6) (HMP Unified Metabolic Analysis Network) pipeline [33]. Within this pipeline, the reads were aligned against the Integrated Microbial Genomes and Microbiomes (IGC2) catalog or the KEGG Orthology (KO) database to quantify the functional capacity of the microbial populations, such as the abundance of KEGG pathways.

### 2.10. Non-Targeted Metabolomics Analysis

Non-targeted metabolomics was performed on serum, liver, and colon samples. Serum metabolites were extracted using ice-cold 80% methanol (1:4, *v*/*v*) for protein precipitation, followed by vortexing, incubation on ice, centrifugation, dilution to 53% methanol, and recentrifugation. Colon and liver tissues were flash-frozen, crushed, and extracted with 80% methanol following the same procedure. A Vanquish UHPLC-Hypersil Gold column coupled to an Orbitrap Q Exactive HF-X (Thermo) provided chromatographic separation and MS detection in positive and negative ion modes. Following processing in Compound Discoverer 3.3, the metabolites were annotated via MS^2^ spectral matches to KEGG and HMDB. Features whose CV was >30% across the QC samples were filtered out. PCA/OPLS-DA identified differentially abundant metabolites, and MetaboAnalyst 6.0 was used to perform pathway enrichment [25,28].

### 2.11. Statistical Analysis

Statistical comparisons were carried out with SPSS 25.0. One-way ANOVA was used for multiple-group analyses, while an independent samples *t* test was applied when only two groups were compared. For post hoc analysis, Tukey’s HSD test was used for multiple comparisons. All data were tested for normality using the Shapiro–Wilk test, and *p* < 0.05 was taken as the threshold for significance.

## 3. Results

### 3.1. Rha Alleviates Clinical Symptoms and Inflammation in Chronic Colitis Mice

**Figure 1:**
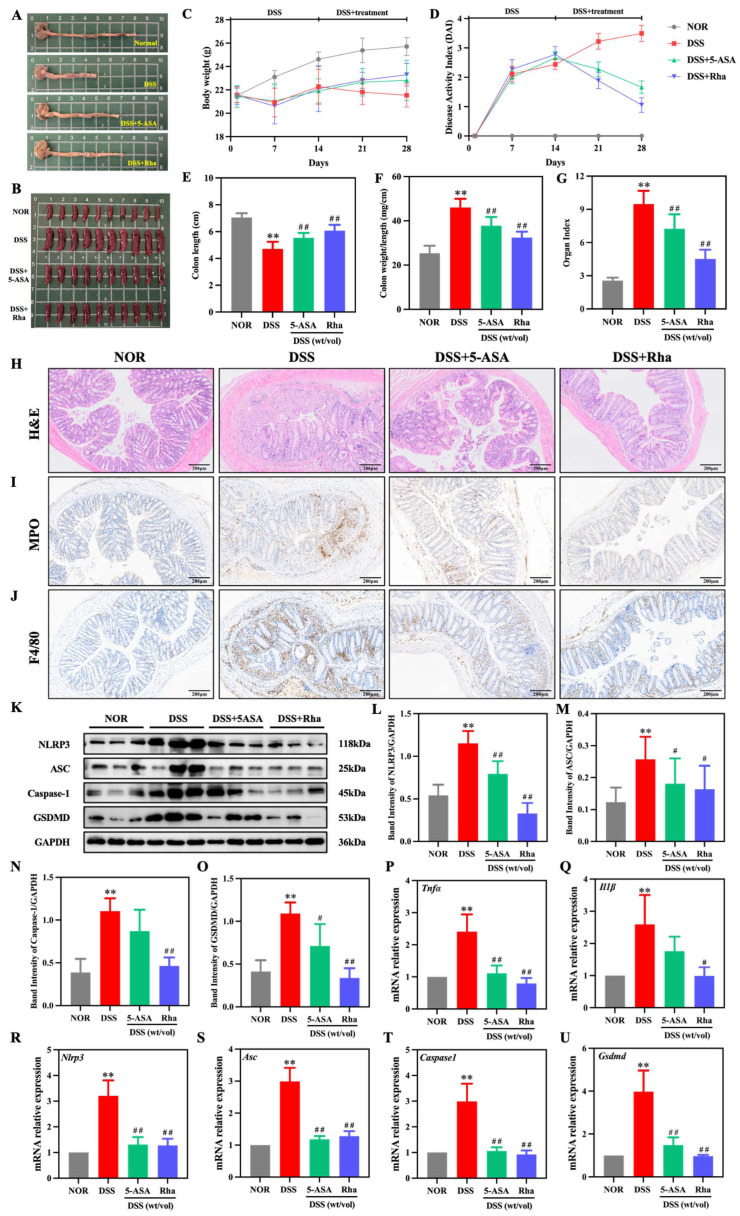
Rha alleviated clinical symptoms and attenuated intestinal inflammation in DSS-induced colitis mice. (**A**) Colon pictures. (**B**) Spleen pictures. (**C**) Mouse body weight change curve. (**D**) DAI score. (**E**) Colon length. (**F**) Colonic index (ratio of colonic weight to length). (**G**) Spleen organ index. (**H**) H&E staining of colon tissues. (**I**,**J**) IHC observation of MPO and F4/80 in the colon. (**K**–**O**) Protein immunoblotting to detect protein expression of NLRP3, ASC, Caspase-1, and GSDMD in the colon. (**P**–**U**) qPCR analysis of *Tnfα*, *Il1β*, *Nlrp3*, *Asc*, *Caspase1*, and *Gsdmd* in mouse colon tissues. Statistical significance is indicated as follows: ** *p* < 0.01 for DSS group vs. NOR group; ^#^ *p* < 0.05, ^##^ *p* < 0.01 for treatment groups vs. DSS group.

Initial body weight was not significantly different among the groups at the beginning of the study. Mice treated with DSS displayed sustained weight loss and a significant increase in DAI. Both the 5-ASA and Rha interventions markedly attenuated these changes, reducing the DAI score and mitigating the weight loss caused by DSS (Figure 1C,D). Furthermore, Rha treatment significantly ameliorated DSS-induced clinical alterations, including colon shortening and increased the colon coefficient and spleen index (Figure 1B–G). H&E staining revealed an intact colon in the NOR test but severe DSS-induced mucosal injury, with inflammatory infiltration, crypt loss, and barrier disruption. These pathological changes were significantly alleviated by both 5-ASA and Rha treatment (Figure 1H). Additionally, the pronounced DSS-induced increase in F4/80 and MPO expression in colon tissue were markedly suppressed by 5-ASA and Rha (Figure 1I,J). MPO^+^ cell area in the DSS group was markedly increased to 21.01-fold compared with the NOR group. Compared with the DSS group, MPO^+^ cell area was reduced by 48.56% in the DSS+5-ASA group and by 85.18% in the DSS+Rha group (Figure S2), indicating that Rha exhibited superior therapeutic effects in reducing MPO^+^ cell infiltration. Compared with NOR, DSS increased the protein expression of NLRP3, ASC, Caspase-1, and GSDMD in colon tissue by 2.13-, 2.08-, 2.85-, and 2.64-fold, respectively (Figure 1K–O). Compared with DSS treatment, Rha treatment resulted in the most pronounced suppression, reducing NLRP3, ASC, Caspase-1, and GSDMD expression by 71.59%, 36.37%, 58.01%, and 69.11%, respectively. Consistently, compared with the NOR group, the DSS group had significantly upregulated mRNA expression of *Nlrp3*, *Asc*, *Caspase1*, *Gsdmd*, *Tnfα*, and *Il1β* in the colon (increased by 2.21-, 1.99-, 1.98-, 2.97-, 1.41-, and 1.59-fold, respectively). Rha intervention significantly reversed these increases (Figure 1P–U), leading to decreases of 60.19%, 57.20%, 69.16%, 75.42%, 67.07%, and 61.79% in the respective mRNA levels in the DSS+Rha group relative to those in the DSS group. Collectively, these findings suggest that Rha alleviates DSS-induced intestinal inflammation by blocking NLRP3 inflammasome activation.

### 3.2. Rha Improves Intestinal Barrier Function in Chronic Colitis Mice by Increasing Mucus Secretion and TJ Protein Expression

**Figure 2:**
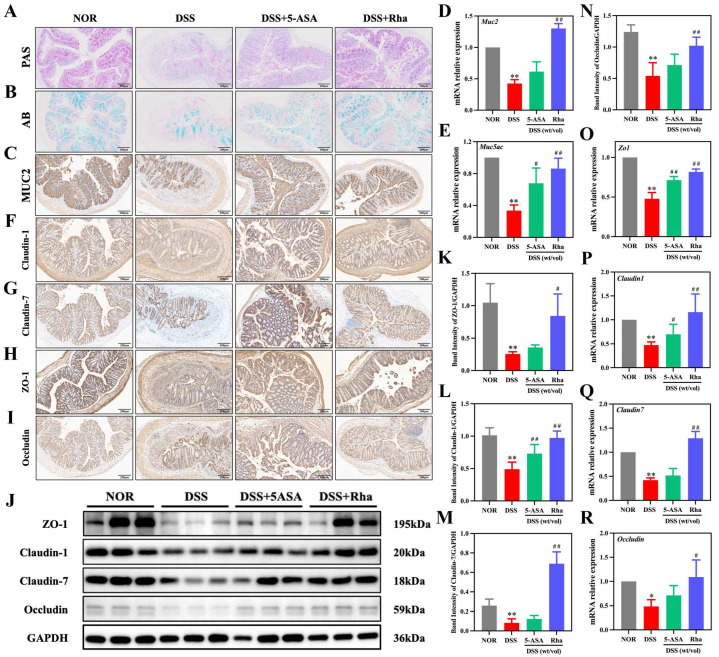
Rha restores intestinal barrier integrity in DSS-induced colitis by enhancing mucus production and TJ expression. (**A**) PAS staining of colon tissues. (**B**) AB staining of colon tissues. (**C**) IHC detection of MUC2 expression in colon tissues. (**D**,**E**) qPCR analysis of *Muc2* and *Muc5ac* mRNAs in colon tissues. (**F**–**I**) IHC observation of Claudin-1, Claudin-7, ZO-1 and Occludin in the colon tissues. (**J**–**N**) Protein levels of ZO-1, Claudin-1, Claudin-7 and Occludin in colon tissues. (**O**–**R**) qPCR analysis of *Zo1*, *Claudin1*, *Claudin7* and *Occludin* mRNAs in colon tissues. Statistical significance is indicated as follows: * *p* < 0.05, ** *p* < 0.01 for DSS group vs. NOR group; ^#^ *p* < 0.05, ^##^ *p* < 0.01 for treatment groups vs. DSS group.

Rha intervention maintained the acidic mucin content (Figure 2A) and protected goblet cells (Figure 2B). Furthermore, compared with DSS treatment, Rha treatment increased the histological abundance of MUC2 (Figure 2C) and upregulated *Muc2* (2.07-fold) and *Muc5ac* (1.57-fold) levels (Figure 2D,E). IHC staining revealed that the abundance of TJ proteins was significantly lower in the DSS group than in the NOR group, and this reduction was markedly reversed by Rha treatment (Figure 2F–I). Protein expression analysis revealed that the expression levels of ZO-1, Claudin-1, Claudin-7, and Occludin were greater in the DSS+Rha group than in the DSS group, with increases of 2.29-, 1.08-, 7.28-, and 1.02-fold, respectively. However, the DSS+5-ASA group exhibited a significant increase in only Claudin-1 (1.45-fold), with no significant changes observed for ZO-1, Claudin-7, or Occludin (Figure 2J–N). At the mRNA level, DSS treatment led to reductions of 52.18%, 52.61%, 57.98%, and 51.36% in *Zo1*, *Claudin1*, *Claudin7*, and *Occludin* levels, respectively. Rha treatment significantly upregulated their mRNA levels by 0.71-, 1.45-, 2.07-, and 1.24-fold. The 5-ASA group showed marked increases in the expression of *Zo1* and *Claudin1* (49.23% and 48.72%, respectively) but not in the expression of *Claudin7* or *Occludin* (Figure 2O–R).

### 3.3. Rha Suppresses Intestinal Fibrosis and Activates Autophagy in UC Mice

**Figure 3:**
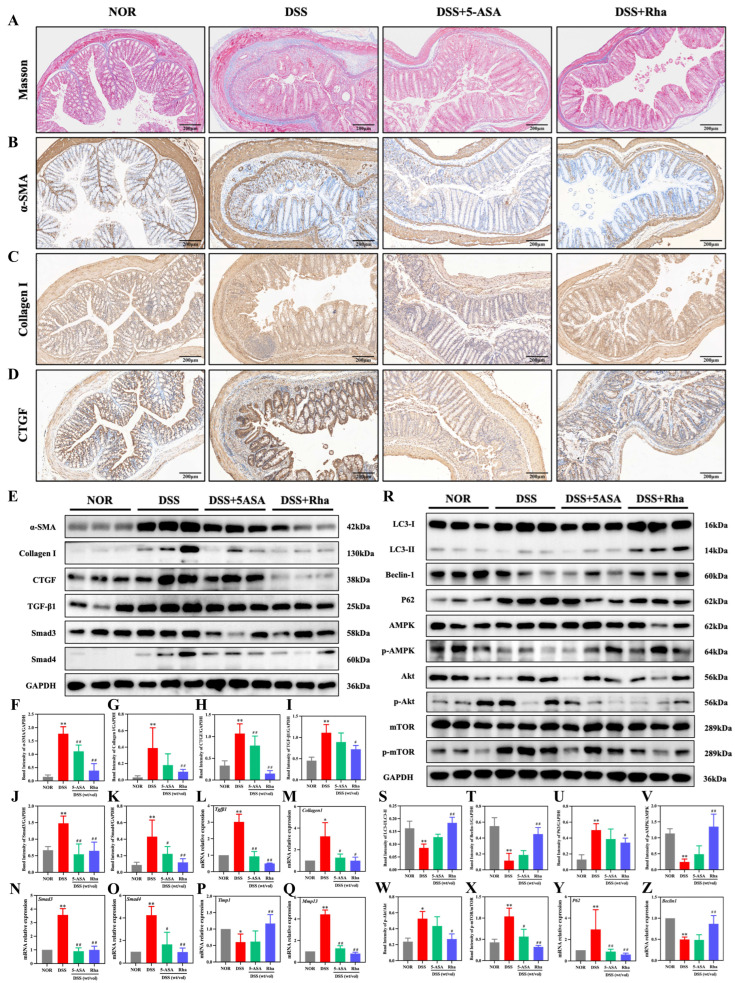
Rha alleviates DSS-induced colitis via activation of autophagy and suppression of fibrosis. (**A**) Masson staining of colon tissues. (**B**–**D**) IHC observation of α-SMA, Collagen I, and CTGF in colon tissues. (**E**–**K**) Protein levels of α-SMA, Collagen I, CTGF, TGF-β1, Smad3, and Smad4 in colon tissues. (**L**–**Q**) qPCR analysis of *Tgfβ1*, *CollagenI*, *Smad3*, *Smad4*, *Timp1*, and *Mmp13* mRNAs in colon tissues. (**R**–**X**) Protein levels of LC3-I/LC3-II, Beclin-1, P62, AMPK/p-AMPK, Akt/p-Akt, and mTOR/p-mTOR in colon tissues. (**Y**,**Z**) qPCR analysis of *P62* and *Beclin1* mRNAs in colon tissues. Statistical significance is indicated as follows: * *p* < 0.05, ** *p* < 0.01 for DSS group vs. NOR group; ^#^ *p* < 0.05, ^##^ *p* < 0.01 for treatment groups vs. DSS group.

Masson staining demonstrated large-scale collagen deposition in DSS-treated mice, suggesting strong fibrosis. Both 5-ASA and Rha significantly alleviated these pathological changes, and Rha was more effective (Figure 3A). Compared with those in the NOR group, the protein expression of α-SMA, Collagen I, and CTGF in the DSS+5-ASA and DSS+Rha groups significantly increased (Figure 3B–D). In protein expression analysis, compared with the NOR group, DSS treatment clearly increased the expression of key profibrotic factors such as α-SMA, Collagen I, CTGF, TGF-β1, Smad3, and Smad4 by 10.52-, 11.51-, 2.23-, 1.45-, 1.22-, and 3.84-fold, respectively. Compared with DSS group, 5-ASA treatment reduced the protein expression of α-SMA, CTGF, Smad3, and Smad4 by 37.21%, 25.97%, 63.32%, and 48.09%, respectively. But Rha treatment more effectively reduced the protein expression of α-SMA, Collagen I, CTGF, TGF-β1, Smad3, and Smad4 by 78.19%, 74.20%, 86.00%, 35.04%, 56.21%, and 72.11%, respectively (Figure 3E–K). On the mRNA scale, qPCR analysis revealed that DSS exposure markedly increased the levels of *Tgfβ1*, *Collagen I*, *Smad3*, *Smad4*, and *Mmp13* by 2.02-, 2.24-, 2.57-, 3.24-, and 3.43-fold, respectively, while Timp1 expression decreased by 39.71%. Rha intervention largely reversed these changes, reducing the mRNA levels of *Tgfβ1*, *Collagen I*, *Smad3*, *Smad4*, and *Mmp13* by 83.54%, 69.54%, 71.96%, 77.18%, and 81.95%, respectively, and increasing *Timp1* mRNA expression by 1.04-fold. Compared with DSS+5-ASA group, Rha intervention reduced the mRNA levels of *Tgfβ1*, *Collagen I*, *Smad4*, and *Mmp13* by 46.70%, 22.31%, 41.43% and 39.24%, respectively, and increasing *Timp1* mRNA expression by 1.52-fold (Figure 3L–Q). The role of autophagy was subsequently explored. Western blot analysis demonstrated that compared with NOR treatment, DSS treatment resulted in a 47.29% reduction in the LC3-II/LC3-I ratio and a 78.79% reduction in Beclin-1 expression, but it increased P62 protein expression by 1.47-fold. However, compared with DSS treatment, Rha treatment effectively reversed these changes, resulting in an increase in the LC3-II/LC3-I ratio by 60.5% and Beclin-1 expression by 93.5% but a decrease in P62 protein expression by 91.45% (Figure 3R–X), suggesting increased autophagic flux. To further explore how autophagy was induced in our model, the role of the AMPK-Akt/mTOR pathway was investigated. Compared with NOR treatment, DSS treatment increased Akt phosphorylation by 1.22-fold but suppressed AMPK phosphorylation by 78.57%, while it increased mTOR phosphorylation by 1.42-fold. These changes could be effectively alleviated by treatment with Rha, which suppressed the phosphorylation of both Akt and mTOR by 48.62% and 68.82%, respectively, but it also increased the phosphorylation of AMPK by 4.54-fold compared with DSS treatment. These data obtained from protein analyses further support the results of the qPCR analysis, which revealed that treatment with Rha suppressed the DSS-induced changes in the expression of genes related to autophagy (*P62* and *Beclin1*). These findings indicate that Rha attenuates intestinal fibrosis and enhances autophagy, likely by regulating AMPK-Akt/mTOR signaling, to protect against chronic UC.

### 3.4. Rha Restores Gut Microbial Composition and Reveals Microbiota–Host Phenotypic Correlations in Chronic UC Mice

**Figure 4:**
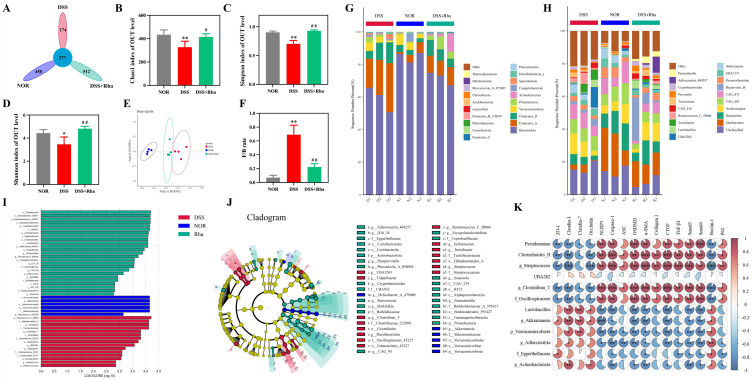
Rha ameliorates intestinal flora disorders in UC mice. (**A**) Petal diagram; (**B**–**D**) Chao1, Simpson and Shannon indices; (**E**) PCoA chart; (**F**) F/B ratio (Statistical significance is indicated as follows: * *p* < 0.05, ** *p* < 0.01 for DSS group vs. NOR group; ^#^ *p* < 0.05, ^##^ *p* < 0.01 for DSS+Rha group vs. DSS group); (**G**) phylum level bar stacking plot; (**H**) genus level bar stacking plot; (**I**,**J**) LEfSe analysis; (**K**) correlation between intestinal flora and host factors (Red indicates positive correlation, whereas blue indicates negative correlation. Statistical significance is indicated as follows: * *p* < 0.05, ** *p* < 0.01, and *** *p* < 0.001).

Because it was superior to 5-ASA in terms of barrier protection and inflammation inhibition, 16S rRNA sequencing was conducted in the NOR, DSS, and DSS+Rha groups. In these groups, there were 1421 OTUs in total, with 458, 174, and 512 OTUs in the NOR, DSS, and DSS+Rha groups, respectively (Figure 4A). After Rha treatment, the number of OTUs dramatically increased and the alpha diversity increased, as shown by the Chao1, Shannon, and Simpson indices (Figure 4B–D). In the analysis using the PCoA plot, there was a clear separation in the clusters of the three groups in terms of the composition of microbes (Figure 4E). The F/B ratio significantly decreased by 67.76% compared with that in the DSS model after Rha treatment. In addition, Rha treatment appeared to profoundly reduce potentially pathogenic bacteria, such as *Pseudomonas*, *Clostridioides_H*, and *UBA282*—whose abundances increased in the DSS treatment model—while increasing the levels of beneficial bacteria such as *Akkermansia* and *Lactobacillus* to restore microbial balance (Figure 4F–H). Regarding the changes in microbes, as determined using LEfSe (LDA > 4), *g_Akkermansia*, *p_Verrucomicrobiota*, and *c_Verrucomicrobia* were enriched in the NOR group; f_Eggerthellaceae, g_Adlercreutzia, and p_Actinobacteriota were enriched in the DSS+Rha group; and *f_Streptococcaceae*, *g_Clostridium_T*, *o_Clostridiatess*, *g_Streptococcus*, and *f_Oscillospiraceae* were enriched in the DSS model (Figure 4J,K). Spearman’s correlation analysis linked these microbial changes to host phenotypes. Inflammation-associated taxa (*Pseudomonas*, *Clostridioides_H*, *g_Streptococcus*, *g_Clostridium_T*, and *f_Oscillospiraceae*) were negatively correlated with TJ proteins and Beclin-1 (*p* < 0.05) but positively correlated with inflammatory, pyroptotic, and fibrotic mediators, such as NLRP3, Caspase-1, ASC, GSDMD, α-SMA, Collagen I, CTGF, TGF-β1, Smad3, Smad4, and P62 (*p* < 0.01). In contrast, inverse correlation patterns were detected for beneficial taxa (e.g., *g_Akkermansia*, *Lactobacillus*, *g_Adlercreutzia*) (*p* < 0.05), emphasizing the protective effect of these taxa.

### 3.5. Effects of Rha on the Serum Untargeted Metabolome in DSS-Induced UC Mice

**Figure 5:**
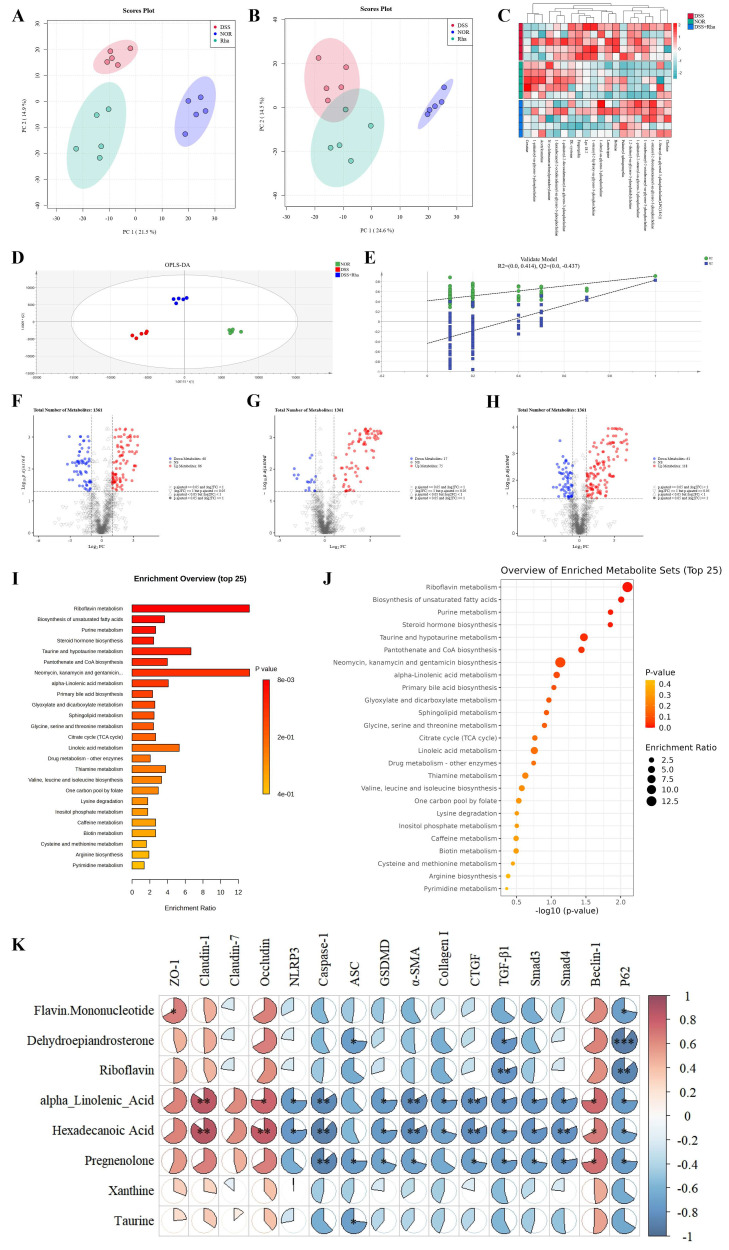
Effects of Rha on serum non-targeted metabolomics in DSS-induced UC mice. (**A**,**B**) PCA analysis diagram in negative ion mode and positive ion mode. (**C**) Heat map of differential metabolites across groups. (**D**) Plot of OPLS-DA scores in positive ion mode. (**E**) OPLS-DA cross-validation plot. (**F**) Plot of differential metabolite screening results between NOR and DSS groups. (**G**) Plot of differential metabolite screening results between DSS and DSS+Rha groups. (**H**) Plot of differential metabolite screening results between NOR and DSS+Rha groups. (**I**,**J**) KEGG enrichment analysis and screening for differential metabolite enrichment pathway bubble diagrams. (**K**) Serum metabolites correlation with host phenotypes (Red indicates positive correlation, whereas blue indicates negative correlation. Statistical significance is indicated as follows: * *p* < 0.05, ** *p* < 0.01, and *** *p* < 0.001).

Non-targeted metabolomic analysis of serum revealed great intragroup reproducibility and clear intergroup separation in the PCA model under positive and negative ion modes (Figure 5A,B), indicating that both UC induction and Rha intervention significantly altered hepatic metabolism. OPLS-DA confirmed significant pairwise metabolic differences (NOR vs. DSS; DSS vs. DSS+Rha), with model validation supporting robustness (Figure 5D,E). Volcano plot visualization revealed that Rha intervention significantly reversed 36 differentially abundant metabolites compared with those in the DSS group (Figure 5F–H). Compared with DSS treatment, Rha treatment notably increased the relative levels of several differentially abundant metabolites, including riboflavin, flavin mononucleotide, hexadecanoic acid, α-linolenic acid (ALA), xanthine, pregnenolone, dehydroepiandrosterone, and taurine. KEGG pathway enrichment (MetaboAnalyst 6.0; impact > 0.1, FDR < 0.05) revealed five significantly altered pathways: riboflavin metabolism, purine metabolism, steroid hormone biosynthesis, unsaturated fatty acid biosynthesis, and taurine and hypotaurine metabolism (Figure 5I,J). We performed Spearman’s correlation analysis of normalized metabolite levels with various phenotypic factors to explore their potential links (Figure 5K). ALA and hexadecanoic acid were positively correlated with Claudin-1, Claudin-7, and Beclin-1 levels (*p* < 0.01). Conversely, these metabolites were significantly negatively correlated with key NLRP3 inflammasome components, the pyroptosis executor GSDMD, intestinal fibrosis markers, and molecules within the TGF-β1/Smad pathway (*p* < 0.05).

### 3.6. Effects of Rha on the Liver Untargeted Metabolome in DSS-Induced UC Mice

**Figure 6:**
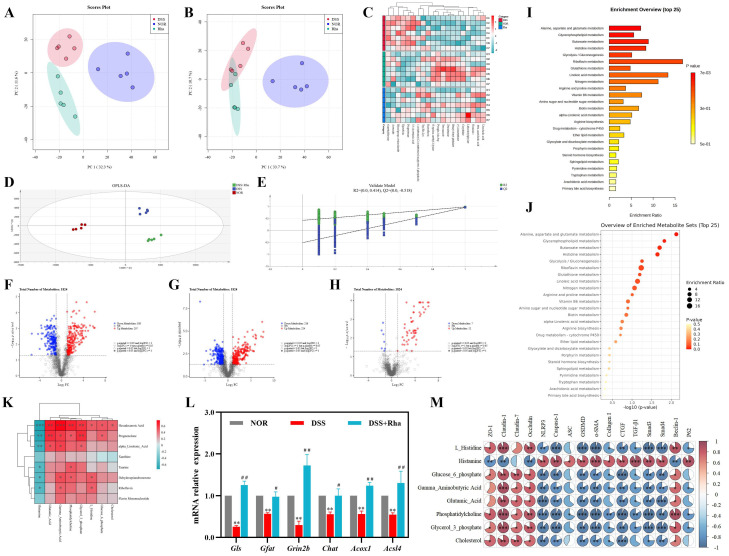
Effects of Rha on hepatic non-targeted metabolomics in DSS-induced UC mice. (**A**,**B**) PCA analysis diagram in negative ion mode and positive ion mode. (**C**) Heat map of differential metabolites across groups. (**D**) Plot of OPLS-DA scores in positive ion mode. (**E**) OPLS-DA cross-validation plot. (**F**) Plot of differential metabolite screening results between NOR and DSS groups. (**G**) Plot of differential metabolite screening results between DSS and DSS+Rha groups. (**H**) Plot of differential metabolite screening results between NOR and DSS+Rha groups. (**I**,**J**) KEGG enrichment analysis and screening for differential metabolite enrichment pathway bubble diagrams. (**K**) Correlation analysis of differential metabolites based on their standardized relative abundance in serum and liver (* *p* < 0.05, ** *p* < 0.01, *** *p* < 0.001). (**L**) mRNA levels of key enzymes in relevant metabolic pathways (Statistical significance is indicated as follows: ** *p* < 0.01 for DSS group vs. NOR group; ^#^ *p* < 0.05, ^##^ *p* < 0.01 for DSS+Rha group vs. DSS group). (**M**) Liver metabolites correlation with host phenotypes (Red indicates positive correlation, whereas blue indicates negative correlation. Statistical significance is indicated as follows: * *p* < 0.05, ** *p* < 0.01, and *** *p* < 0.001).

Non-targeted metabolomic analysis of liver tissue revealed good intragroup reproducibility and clear intergroup separation in the PCA model under positive and negative ion modes (Figure 6A,B), indicating that both UC induction and Rha intervention significantly altered hepatic metabolism. OPLS-DA confirmed significant pairwise metabolic differences (NOR vs. DSS; DSS vs. DSS+Rha), with model validation supporting the robustness of these findings (Figure 6D,E). On the basis of the predefined statistical criteria, we identified differentially abundant metabolites and constructed volcano plots (Figure 6F–H). A total of 207 differentially abundant metabolites were identified between the NOR and DSS groups, 52 of which were significantly reversed toward normal levels by Rha intervention. Specifically, compared with DSS treatment, Rha treatment increased the relative levels of glutamate, phosphatidylcholine, glycerol-3-phosphate, cholesterol, γ-aminobutyric acid (GABA), glucose-6-phosphate, and L-histidine but decreased the level of histamine. Four significantly altered pathways were identified through KEGG pathway enrichment (Impact > 0.1, FDR < 0.05): alanine, aspartate, and glutamate metabolism (map00250); glycerophospholipid metabolism; butanoate metabolism; and histidine metabolism (Figure 6I,J). Correlation analysis between serum and liver metabolomes revealed that serum hexadecanoic acid was negatively correlated with liver histamine and positively correlated with liver glutamate, GABA, phosphatidylcholine, and glycerol-3-phosphate (*p* < 0.01). Additionally, pregnenolone, ALA, riboflavin, dehydroepiandrosterone, and taurine levels were negatively correlated with liver histamine levels but positively correlated with liver GABA and phosphatidylcholine levels (*p* < 0.05; Figure 6K). To further validate the impact of Rha on hepatic metabolism, we quantified the mRNA levels of key metabolic enzymes in the liver. Glutaminase (GLS) is crucial for glutamate generation, and glutamate is a precursor of GABA. Compared with NOR, DSS exposure significantly downregulated the mRNA expression of *Gls*, *Gfat*, *Grin2b*, *Chat*, *Acox1*, and *Acsl4* by 74.16%, 43.19%, 70.06%, 44.26%, 43.87%, and 45.11%, respectively. Compared with DSS treatment, Rha intervention significantly upregulated the expression of these genes by 3.87-, 0.72-, 4.77-, 0.80-, 1.21-, and 1.38-fold, suggesting that Rha promotes the hepatic generation of glutamate and GABA (Figure 6L). Spearman’s correlation analysis between liver metabolites and host phenotypic factors (Figure 6M) revealed that the levels of liver metabolites (including L-histidine, glucose-6-phosphate, GABA, glutamate, phosphatidylcholine, glycerol-3-phosphate, and cholesterol) were markedly positively correlated with those of TJ proteins and the autophagy marker Beclin-1 but negatively correlated with those of inflammatory factors, fibrosis indicators, and the autophagy inhibitor P62 (*p* < 0.01). In contrast, liver histamine levels were negatively correlated with intestinal TJ proteins (Claudin-1, ZO-1, and Occludin) and Beclin-1 (*p* < 0.01) and positively correlated with inflammatory factors, fibrosis indicators, and P62 (*p* < 0.05).

### 3.7. Rha Ameliorates Colonic Metabolic Alterations in UC Mice

**Figure 7:**
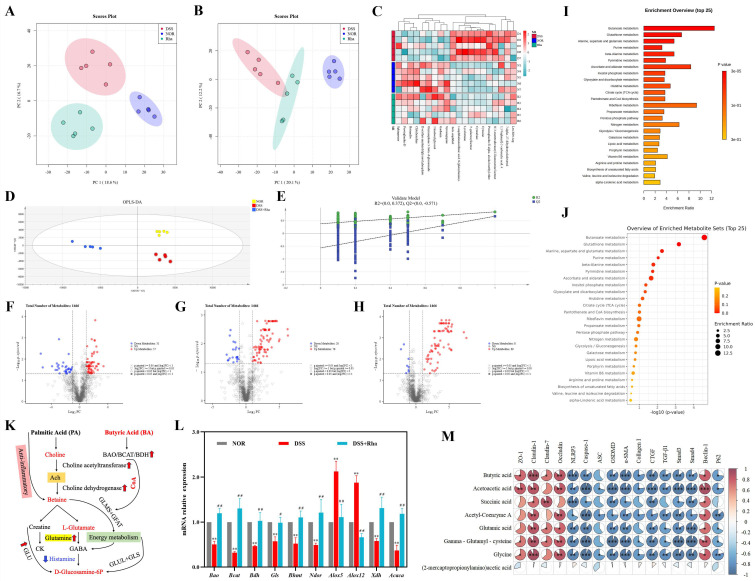
Effects of Rha on colon non-targeted metabolomics in DSS-induced UC mice. (**A**,**B**) PCA analysis diagram in negative ion mode and positive ion mode. (**C**) Heat map of differential metabolites across groups. (**D**) Plot of OPLS-DA scores in positive ion mode. (**E**) OPLS-DA cross-validation plot. (**F**) Plot of differential metabolite screening results between NOR and DSS groups. (**G**) Plot of differential metabolite screening results between DSS and DSS+Rha groups. (**H**) Plot of differential metabolite screening results between NOR and the DSS+Rha groups. (**I**,**J**) KEGG enrichment analysis and screening for differential metabolite enrichment pathway bubble diagrams. (**K**) Diagram of colon tissue metabolic pathway. (**L**) mRNA levels of key enzymes in relevant metabolic pathways (Statistical significance is indicated as follows: ** *p* < 0.01 for DSS group vs. NOR group; ^#^ *p* < 0.05, ^##^ *p* < 0.01 for DSS+Rha group vs. DSS group). (**M**) Colon metabolites correlation with host phenotypes (Red indicates positive correlation, whereas blue indicates negative correlation. Statistical significance is indicated as follows: * *p* < 0.05, ** *p* < 0.01, and *** *p* < 0.001).

Non-targeted PCA of colon tissue (Figure 7A,B) revealed distinct separation between the NOR, DSS, and DSS+Rha groups in positive and negative ion modes, indicating that UC induction and Rha intervention significantly altered the metabolic profiles. Subsequent permutation tests confirmed that the constructed OPLS-DA models were robust and reliable and did not suffer from overfitting. A total of 432 positive-ion metabolites and 378 negative-ion metabolites were identified. Differentially abundant metabolites were then screened using VIP > 1, FC > 1.2 or <0.8, and *p* < 0.05 and visualized in volcano plots. Compared with the NOR group, the DSS group had 57 upregulated and 31 downregulated metabolites. Compared with the DSS group, the DSS+Rha group presented 78 metabolites whose levels increased and 20 whose levels decreased. Notably, 23 metabolites overlapped between these comparisons. MetaboAnalyst 6.0 (KEGG 2022, impact > 0.1, FDR < 0.05) identified butanoate metabolism (map00650), glutathione metabolism, and alanine/aspartate/glutamate as the top three modulated pathways (Figure 7I,J). We then quantified six key differentially abundant metabolites within these pathways. Compared with the DSS group, the DSS+Rha group showed marked increases in butyric acid, acetoacetic acid, succinic acid, acetyl-CoA, glutamate, and γ-glutamylcysteine levels. To trace metabolic interplay across tissues, we constructed a network map based on the relationships between significant metabolites, illustrating their flow and interactions among serum, liver, and colon tissues (Figure 7K). Finally, we assessed the colonic mRNA expression of key metabolic enzymes in these pathways (Figure 7L). Compared with the NOR group, the DSS group had significantly lower expression levels of *Bao*, *Bcat*, *Bdh*, *Gls*, *Bhmt*, *Ndor*, *Xdh*, and *Acaca* (49.52%, 67.94%, 53.49%, 42.84%, 47.87%, 50.97%, 42.08%, and 63.05%, respectively). In contrast, the levels of *Alox5* and *Alox12* significantly increased by 1.13-fold and 0.87-fold, respectively. Rha intervention significantly reversed these changes, increasing the mRNA levels of *Bao*, *Bcat*, *Bdh*, *Gls*, *Bhmt*, *Ndor*, *Xdh*, and *Acaca* by 1.37-, 3.07-, 1.21-, 0.73-, 1.16-, 1.47-, 1.27-, and 2.21-fold, respectively, and decreasing the levels of *Alox5* and *Alox12* by 48.12% and 64.59%, respectively, compared with those in the DSS group. Spearman’s correlation analysis between colonic metabolites and host phenotypic factors (Figure 7M) revealed that a series of metabolites, including butyric acid, acetoacetic acid, succinic acid, acetyl-CoA, glutamate, γ-glutamylcysteine, and glycine, exhibited coordinated changes with the phenotypes. These metabolites were significantly positively correlated with the expression of TJ proteins and the autophagy marker Beclin-1 (*p* < 0.05) and negatively correlated with the expression of inflammatory factors, fibrosis markers, and P62 (*p* < 0.01).

### 3.8. Metagenomic and Targeted Metabolomic Analyses Reveal the Impact of Rha on the Gut Microbiota and SCFA Production

**Figure 8:**
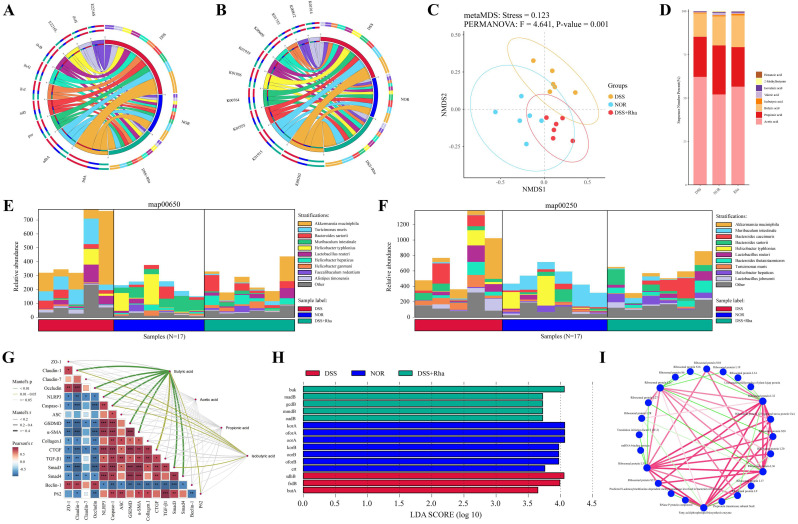
Rha regulates the fecal metagenome in DSS-induced UC mice and promotes SCFAs production. (**A**) Circos analysis of metagenomic data. (**B**) Circos plot of KEGG Orthology (KO) abundance. (**C**) NMDS plot of microbial community structure. (**D**) Species stratification analysis based on map00650. (**E**) Species stratification analysis based on map00250. (**F**) SCFAs content. (**G**) Mantel tests of SCFAs, inflammation-related factors, TJ proteins, fibrosis-related factors, and autophagy-related factors (* *p* < 0.05, ** *p* < 0.01, *** *p* < 0.001). (**H**) LEfSe analysis showing differentially enriched features. (**I**) Protein–protein interaction network topology analysis.

As shown in Figure 8, Circos analysis of our fecal metagenomic data indicated that Rha effectively reversed the functional imbalance of the gut microbiota induced by DSS. Specifically, compared with DSS, Rha intervention significantly increased the relative abundances of genes involved in branched-chain amino acid synthesis (e.g., *ilvH* and *ilvB*) and tricarboxylic acid cycle/anaerobic respiration (e.g., *sdhA* and *frdA*) (Figure 8A). Furthermore, the results of the KO analysis revealed that Rha significantly modulated key functional units related to SCFA synthesis. The expression level of the acetyl-CoA carboxylase gene (K01914), which represents the first essential step in SCFA synthesis, returned to baseline after Rha treatment (Figure 8B). NMDS analysis using the Bray–Curtis distance measure effectively demonstrated the distinct differences in microbial structural diversity among the DSS, NOR, and Rha treatment categories (Figure 8C). The resulting ordination presented an optimal model fit (stress = 0.123) with a proper representation of the actual sample distances. PERMANOVA further eliminated any doubts concerning the existence of significant intergroup differences in microbial diversity (F = 4.641, *p* = 0.001). Species distribution mapping using functional categories 00650 and 00250 revealed that the disruption in taxonomical members of these functional categories was profoundly impacted by DSS-induced colitis. However, the Rha treatment tended to restore these species to a level closer to that in NOR-treated mice, suggesting normalization of microbiome-associated metabolic functions (Figure 8E,F). In these two functional categories, the most dramatic change was found in the proportional representation of functional genes related to *A. muciniphila*. This trend reflects that Rha treatment chiefly affected these metabolic networks in accordance with changes in the functional contributions of *A. muciniphila*, which agreed with the results obtained from the 16S rRNA analysis. Metabolomic analysis further demonstrated that the level of butyrate was drastically reduced in the DSS-treated mice; however, Rha treatment restored the level of butyrate close to the basal standards (Figure 8D). Mantel test analysis revealed significant associations between butyrate availability and host phenotypes related to barrier integrity, inflammation, fibrosis, and autophagy (Mantel’s r ≥ 0.4; *p* < 0.05). Isobutyric acid displayed a similar albeit weaker correlation pattern. LEfSe analysis further revealed significant enrichment of the butyrate synthesis gene *buk* following Rha intervention (LDA > 4; Figure 8H), and this functional shift was supported by protein–protein interaction analysis (Figure 8I).

## 4. Discussion

UC is characterized by chronic and persistent inflammation in the intestines due to damage to the intestinal barrier, persistent inflammation, and fibrosis in tissues [34]. Our previous findings indicated that Rha effectively suppressed inflammation in the intestines and facilitated the repair of the barrier in mice with acute DSS-induced colitis [25]. However, in the present study, Rha effectively increased the length of the colon, decreased the DAI and spleen index, and suppressed pro-inflammatory cytokines to effectively alleviate chronic colitis in mice induced by DSS for 28 days. NLRP3 inflammation is highly prevalent in the intestines and facilitates the cleavage of GSDMD in the process of intestinal pyroptosis triggered by inflammation [35]. Here, we revealed that Rha effectively suppressed the activation of NLRP3 inflammation by reducing the expression of essential components (GSDMD, Caspase-1, and ASC) and further suppressed the secretion of pro-inflammatory cytokines (TNF-α and IL-1β). An impaired barrier is characterized by damage to the expression of TJ proteins and MUC2 in the tissues affected by UC [36]. In our study, Rha effectively alleviated barrier damage by upregulating the protein expression of MUC2 and MUC5AC in the colon and increasing the expression of the main TJ proteins.

Myofibroblast hyperactivation and excessive ECM deposition are key drivers of intestinal fibrosis [37]. Rha treatment attenuated the DSS-induced upregulation of fibrosis-associated proteins (α-SMA, Collagen I, fibronectin, and TGF-β1) and reduced collagen deposition in our chronic UC model, which is consistent with our previous report [23]. Since impaired autophagy promotes fibrosis through myofibroblast activation and compromised ECM degradation [38], we assessed this process. Chronic colitis is known to suppress autophagy, characterized by decreased Beclin-1 levels and LC3-II/I ratios along with P62 accumulation [39,40]. In our study, Rha markedly increased Beclin-1 levels and LC3-II/I ratios while reducing P62 levels, supporting the use of autophagy restoration to treat colitis-associated fibrosis [41]. An imbalance between the autophagy suppressor Akt/mTOR and the activator AMPK underlies autophagy suppression and impaired immune-inflammatory responses [42]. Our data confirmed that DSS increased Akt and mTOR phosphorylation but suppressed AMPK phosphorylation, indicating impaired autophagy. Rha intervention reversed these changes, which is consistent with reports that natural phytochemicals can alleviate intestinal fibrosis by restoring autophagy [20,27]. Autophagy is essential for preserving gut stability and maintaining barrier integrity. The integrity of the intestinal barrier is essential for blocking harmful gut bacteria and preventing their translocation, which induces inflammatory responses. Therefore, we further investigated the regulatory effect of Rha on the gut microbiota in mice with colitis.

Gut microbiota dysbiosis is a hallmark of UC. Rha intervention effectively reversed the reduction in gut microbial alpha diversity induced by DSS, as shown by 16S rRNA sequencing. Rha treatment significantly decreased the F/B ratio, which is positively correlated with intestinal inflammation levels [43]. Notably, transplantation of gut microbiota with a high F/B ratio induces the expansion of Th1/Th17 cells and increases IFN-γ/IL-17A expression in the lamina propria, exacerbating colitis in germ-free mice [44]. Furthermore, Rha intervention promoted the proliferation of several beneficial bacteria, including *Akkermansia muciniphila* (*A. muciniphila*) and *Lactobacillus*. *A. muciniphila*, an intestinal mucus-foraging commensal, is a sensitive barometer of gut barrier integrity [45]. This species enhances goblet cell function, promotes mucus layer thickening, and increases the expression of TJ proteins via the production of SCFAs, thereby protecting the intestinal epithelial barrier [46,47]. The degradation of mucins by *A. muciniphila* can upregulate the expression of the goblet cell proliferation marker Ki67 and the transcription of MUC2, the primary component of the intestinal mucus layer, reinforcing barrier function [48]. We observed positive correlations between fecal butyrate levels, the relative abundances of *Akkermansia* and *Lactobacillus*, and the levels of TJ proteins in UC model mice (Figure S1). *A. muciniphila* also utilizes intestinal mucus, releasing oligosaccharides and acetate that butyrate-producing organisms such as *Faecalibacterium prausnitzii* can convert into butyrate, a metabolite known for its anticolitic properties. In vitro coculture of *A. muciniphila* with *Faecalibacterium prausnitzii* increased butyrate production [49]. Butyrate-producing bacteria such as *Faecalibacterium prausnitzii* convert acetate to butyrate via the acetyl-CoA condensation pathway, thereby stabilizing the gut microbial community and enhancing anti-inflammatory capacity [50]. *Lactobacillus* strains alleviate intestinal inflammation through multiple mechanisms. Their cell wall component LTA stimulates IL-10 and concurrently downregulates IL-6 and TNF-α [51]. Exopolysaccharides (EPSs) produced by Lactobacillus promote barrier repair, increase SCFA metabolism, upregulate anti-inflammatory mediator expression, and modulate immune cell function, collectively maintaining microbial homeostasis and exerting anti-inflammatory effects [52,53]. Our association analysis identified *Pseudomonas* and *Clostridioides_H* as potential detrimental taxa. The relative abundances of these genera negatively correlated with the expression of intestinal TJ proteins and Beclin-1 but showed positive correlations with NLRP3 inflammasome activation, pyroptosis, and fibrosis markers (α-SMA and CTGF; *p* < 0.05). These results may be linked to the fact that *Pseudomonas aeruginosa* ExoS destabilizes the enterocyte cytoskeleton and redistributes tight junction proteins to breach barrier function [54], whereas *Clostridioides difficile* TcdB activates the NLRP3 inflammasome to trigger epithelial pyroptosis [55]. In contrast, the abundances of bacteria such as *Lactobacillus* and *Akkermansia* were significantly positively correlated with increased intestinal barrier integrity and autophagy activity while negatively correlated with the levels of fibrotic and inflammatory factors, indicating protective effects. Specific strains such as *Lactobacillus reuteri* and *Lactobacillus rhamnosus* GG consolidate the barrier by upregulating the expression of ZO-1 in intestinal cells [56]. *A. muciniphila* not only increases mucus layer thickness but also produces SCFAs to improve barrier integrity [57]. LEfSe analysis further revealed enrichment of *f_Eggerthellaceae* and *g_Adlercreutzia* following Rha intervention (Figure 4K). The abundance of taxa such as Eggerthellaceae and *Adlercreutzia* has been associated with the metabolic transformation of phytochemicals and protection against diseases [58,59].

Non-targeted metabolomics of colon tissue revealed a significant increase in butyrate levels after Rha intervention, which aligned with our targeted fecal SCFA assays. This finding points to a microbial origin for the elevated butyrate concentration. Butyrate kinase (*buk*) is an established marker of microbial butyrate synthesis capacity [60]. In our data, Rha led to a significant enrichment of the *buk* gene in the fecal metagenomes (Figure 8H). Together with the observed increase in colonic butyrate, these findings indicate that Rha enhances the inherent capacity of the gut microbiota to produce butyrate. Compared with those in the DSS-treated group, the critical enzymes (*Bao*, *Bcat*, and *Bdh*) involved in the butyrate metabolic pathway in the colon in the DSS+Rha group were upregulated. This upregulation promotes butyrate production in the colon and increases the uptake and oxidation of butyrate in intestinal cells, as shown by Yang et al. [61]. Correlation analysis between the gut microbiota and colonic metabolites under Rha intervention indicated that a greater abundance of the key beneficial bacterium Akkermansia was positively correlated with increased colonic butyrate and hepatic GABA levels (Figure S1), suggesting that *Akkermansia* may be a pivotal functional bacterium driving this beneficial metabolic network. The results of the Mantel test confirmed that butyrate was one of the metabolites most strongly associated with phenotypic improvement (Figure 8G, *p* < 0.01). It was positively associated with Claudin-1 and Occludin but negatively associated with NLRP3, GSDMD, P62, and key fibrotic molecules (α-SMA and CTGF; *p* < 0.01). As a histone deacetylase inhibitor, butyrate can inhibit NF-κB activity, reducing NLRP3 inflammasome-related pyroptosis. It also activates GPR109A, promoting the expression of ZO-1 to reinforce the epithelial barrier [62,63]. Cai et al. reported that butyrate and its derivatives promote AMPKThr172 phosphorylation and inhibit mTORSer2448 phosphorylation, thereby activating AMPK/mTOR-mediated autophagy [64]. Additionally, butyrate significantly reduces collagen deposition by regulating macrophage M1/M2 polarization, suppressing fibroblast proliferation, and promoting their apoptosis [65]. Disruption of the intestinal barrier in UC mice allows pathogen-associated LPS to reach the liver through mesenteric and portal circulation, activating the hepatic TLR4/MyD88/NF-κB pathway and causing liver injury [66]. DSS-induced colitis mice exhibit significantly elevated hepatic LPS levels, along with increased liver enzyme activities (ALP, AST, ALT) and increased levels of IL-6 [67]. Our study indicated that hepatic metabolomic pathway enrichment analysis in Rha-treated UC mice primarily highlighted the map00250 and map00650 pathways. The most prominent features were the activation of the glutamate/GABA metabolic axis and the downregulation of histamine expression. Specifically, Rha intervention significantly increased the relative levels of glutamate, GABA, L-histidine, glycerol-3-phosphate, and phosphatidylcholine but significantly decreased the level of histamine (Figure 6C). We further found that the hepatic metabolites glutamate and GABA were positively associated with intestinal barrier function and autophagy activity factors (*p* < 0.01) but negatively associated with the expression levels of intestinal NLRP3, GSDMD, α-SMA, and CTGF (*p* < 0.01; Figure 6M). Glutamate and GABA reinforce the epithelial barrier by upregulating the expression of TJ proteins, and they stimulate autophagy through GABA_B receptor signaling, as well as through the GCN2/eIF2α/ATF4 and Nrf2/HO-1 axes [68]. These metabolites also dampen inflammation by blocking the TLR4/MyD88/NLRP3 cascade, suppressing Caspase-1 activity and lowering GSDMD expression [69,70]. Correlation analysis between colonic butyrate and hepatic metabolites (Figure S1) revealed that colonic butyrate was negatively correlated with hepatic histamine (*p* < 0.001) but positively correlated with hepatic glutamate and GABA (*p* < 0.001). Colonic and hepatic glutamate levels were positively correlated. These results imply that gut-derived butyrate functions as an upstream regulator, remotely regulating the activation of the hepatic glutamate/GABA metabolic axis via the gut–liver axis to suppress histamine accumulation. Glutamate supplementation effectively alleviated DSS-induced intestinal inflammation by promoting the secretion of IL-4 and IL-10 [71]. Furthermore, Rha intervention notably increased the mRNA levels of key glutamate metabolism enzymes (*Gls* and *Grin2b*) in the livers of UC mice, promoting the synthesis of the immunomodulatory molecule GABA; these findings are consistent with those of Bhat et al. [72]. GABA can also inhibit macrophage activation and pro-inflammatory cytokine production [73]. We further observed a negative correlation between hepatic histamine levels and intestinal TJ proteins (*p* < 0.01), indicating that a reduction in hepatic histamine contributes to the intestinal protective effects of Rha.

The link between butyrate produced in the gut and liver metabolism implies the existence of a local mechanism through which Rha creates an anti-inflammatory environment in the gut–liver axis. These benefits can occur not only in the local context but also in response to systemic cues corresponding to the resolution of inflammation in the body [74]. Serum nontargeted metabolomics revealed that Rha restored circulating ALA levels to normal levels. These findings suggest that Rha may increase the ω-3 PUFA metabolic pathway, thereby supplying the substrates required for the synthesis of SPMs [75,76]. Because ALA is a metabolic precursor to EPA and DHA, alterations in ALA metabolism are closely linked to PUFA-derived endogenous transformations [77,78]. These metabolites reduce neutrophil recruitment and increase macrophage-mediated clearance of apoptotic cells and debris, thereby suppressing inflammatory initiation and promoting tissue repair [79]. Correlation analysis further revealed that ALA levels were positively associated with intestinal barrier proteins and Beclin-1 and negatively associated with NLRP3 activation, pyroptosis, and fibrosis. Studies have shown that ALA and its metabolites EPA and DHA activate the PPARγ pathway to increase the expression of TJ proteins and intestinal barrier integrity [80]. In addition, ω-3 PUFAs increase autophagy via the AMPK-mTOR axis, significantly increasing Beclin-1 expression and facilitating the clearance of damaged organelles [81]. More importantly, ALA inhibits ROS production and NF-κB activation, blocking NLRP3 assembly and Caspase-1-mediated GSDMD cleavage and consequently suppressing IL-1β release and pyroptosis [82]. By inhibiting the TGF-β1/Smad pathway, ALA reduces collagen deposition and the expression of fibrotic factors [83]. Therefore, ALA contributes to the systemic anti-inflammatory, barrier-protective, and antifibrotic effects observed with Rha intervention.

Fecal metagenomic analysis provided functional annotations that complemented the 16S rRNA and metabolomic findings [84,85]. The results confirmed the Rha-induced enrichment of beneficial taxa such as *Akkermansia* and *Lactobacillus* and directly revealed associated metabolic shifts. Critically, the DSS+Rha group showed marked enrichment of *buk* (Figure 8H, LDA > 4). In addition, there was a return to baseline expression levels of genes involved in butyrate synthesis in microbes (Figure 8E), validating the increase in butyrate in the colon and feces. Glutamate metabolic pathway normalization (Figure 8F) in host tissues provided fundamental evidence to support the activation of the gut–liver GABA/glutamate axis. Thus, through metagenomics analysis, functional verification between microbial changes and host metabolic pathways was performed, which helps elucidate how Rha modulates microbiota function and functional benefits through therapy. In conclusion, multiomics analysis revealed that Rha increases the abundance of protective bacteria, such as *A. muciniphila* and *Lactobacillus*, and decreases the abundance of pathogenic bacteria, such as *Pseudomonas* and *Clostridioides_H*, thus leading to increased levels of protective metabolites, such as butyrate, GABA, glutamate, and ALA. These compounds interact in the gut–liver axis network to prevent barrier dysfunction in the intestines, prevent inflammation through the NLRP3 inflammasome and pyroptosis, and reduce fibrosis in tissues through autophagy. However, the role of butyrate in preventing inflammation in tissues was demonstrated through comprehensive multiomics analysis to validate associations in the gut–liver axis in terms of how all these compounds interact to trigger protective changes in tissues. Comprehensive scientific analysis revealed that this network of compounds is fundamental for determining how Rha plays a role in preventing inflammation in tissues.

## 5. Conclusions

In this study, through integrated multiomics analysis, we elucidated the mechanism by which Rha works synergistically to alleviate ulcerative colitis by remodeling the intestinal flora and driving metabolic interactions with butyric acid. These results suggest that Rha intervention inhibits NLRP3-mediated cellular pyroptosis and significantly enhances the functional integrity of the intestinal barrier. Rha also exerts antifibrotic effects by restoring autophagy through activation of the AMPK-Akt-mTOR pathway. Multiomics correlation analyses closely linked the level of bacterial flora with beneficial metabolites derived from different tissues and phenotypic improvement, establishing butyric acid as a functional hub, which synergistically ameliorates intestinal inflammation, repairs the intestinal epithelial barrier, activates autophagy, and inhibits the process of colon fibrosis as the core mechanism underlying the therapeutic effects of Rha.

## Data Availability

All data supporting the findings of this study are included in this published article and its Supplementary Materials. The sequencing data generated during the current study have been deposited in the NCBI Sequence Read Archive (SRA) under BioProject accession number PRJNA1466253 and are publicly available at https://www.ncbi.nlm.nih.gov/bioproject/PRJNA1466253 (accessed on 10 May 2026). Additional datasets analyzed during the current study are available from the corresponding author upon reasonable request.

## Supplementary Materials

The following supporting information can be downloaded at: https://www.mdpi.com/article/10.3390/antiox15050639/s1. Table S1: Disease Activity Index (DAI) scoring criteria. Table S2: Primers sequences of qRT-PCR. Table S3: Primary antibodies of western blot. Figure S1: Correlation Heatmap Analysis of Differential Metabolites and Gut Microbiota Abundance. Figure S2: Immunohistochemical (IHC) semi-quantitative analysis of MPO-positive cells in colon tissue.
