# Supplementary material for: Rhamnocitrin Ameliorates the Intestinal Fibrosis in DSS-Induced Colitis Mice by Modulating Host-Metabolites and Remodeling the Gut Microbiome
Source: Antioxidants (Basel). 2026 May 18;15(5):639. doi: 10.3390/antiox15050639 (PMC13203514; doi:10.3390/antiox15050639)
Supplement: Supplementary file 1 [file antioxidants-15-00639-s001.zip › antioxidants-4280528-supplementary.pdf]

Supplementary Table S1

Disease Activity Index (DAI) scoring criteria

| Indicator | Judgment standard | Score |
|---|---|---|
| Inflammation | Normal | 0 |
| | Mild adhesion | 1 |
| | Severe adhesion | 2 |
| Cytopathic effect | None | 0 |
| | Submucosa | 1 |
| | Muscular layer | 2 |
| | Serosal layer | 3 |
| Degree of crypt damage | Crypt destruction | 0 |
| | 1/3 basement membrane | 1 |
| | 2/3 basement membrane | 2 |
| | Only complete surface epithelium | 3 |
| | All crypts and epithelium are destroyed | 4 |
| Extent of disease | 0 | 0 |
| | 1%–25% | 1 |
| | 26%–50% | 2 |
| | 51%–75% | 3 |
| | 76%–100% | 4 |

Supplementary Table S2

Primers sequences of qRT-PCR

| Primers | Primer sequence (5′-3′) | Primer sequence (3′-5′) | Source |
|---|---|---|---|
| *Gapdh* | AGGTCGGTGTGAACGGATTTG | TGTAGACCATGTAGTTGAGGTCA | Mouse |
| *Nlrp3* | CTATCAAGGACAGGAACG | TAGCTCATCAAAGCCATC | Mouse |
| *Asc* | TCTGGAGTCGTATGGCTTGG | TGCTTGCCTGTGCTGGTC | Mouse |
| *Caspase1* | GAAAGACAAGCCCAAGGT | GTTGAAGAGCAGAAAGCA | Mouse |
| *Gsdmd* | CCATCGGCCTTTGAGAAAGTG | ACACATGAATAACGGGGTTTCC | Mouse |
| *Tnfα* | CTGAACTTCGGGGTGATCGG | GGCTTGTCACTCGAATTTTGAGA | Mouse |
| *Il1β* | AGCACCTTCTTTTCCTTC | TGCCGTCTTTCATTACAC | Mouse |
| *Zo1* | ACCACCAACCCGAGAAGAC | CAGGAGTCATGGACGCACA | Mouse |
| *Claudin1* | GGGGAGACGACAAAGTGA | GCCCAGCCAATAAAGATG | Mouse |
| *Claudin7* | CTGGAGGCATTGTTTTCATTGTG | CATGGGCGTCAAGGGGTTA | Mouse |
| *Occludin* | TTGAAAGTCCACCTCCTTACAGA | CCGGATAAAAAGAGTACGCTGG | Mouse |
| *Muc2* | GCTGACGAGTGGTTGGTGAATG | GATGAGGTGGCAGACAGGAGAC | Mouse |
| *Muc5ac* | TCTACCACTCCCTGCTTCT | TGACTAACCCTCTTGACCAC | Mouse |
| *Tgfβ1* | TACGGCAGTGGCTGAACCAA | CGGTTCATGTCATGGATGGTG | Mouse |
| *Collagen1* | AGACCTGTGTGTTCCCTACT | GAATCCATCGGTCATGCTCTC | Mouse |
| *Smad3* | AGCCGGTGCAGAAAACAGTAA | AGGCGGTCTAACTCTGTGTTC | Mouse |
| *Smad4* | AGTGTGCAGTATAACAAAACGCC | CGTAAAGCAGACTGGGAAGGAAT | Mouse |
| *Timp1* | TGGGGAGATACCACATTTCTGA | TGAGGAAGTCTGAGGGCAATTT | Mouse |
| *Mmp13* | CTGGGCTACACTGAGCACC | AAGTGGTCGTTGAGGGCAATG | Mouse |
| *P62* | TTGCCCATCGAGGACCAGAT | GTCTCCGCGTTGAACACTGT | Mouse |
| *Beclin1* | AGGATGACACCCATCTACCTCAC | CATCGCTCTTCTCAATGAGCA | Mouse |
| *Gls* | GACAACGTCAGATGGTGTCAT | TGCTTGTGTCAACAAAACAATGT | Mouse |
| *Gfat* | GAAGCCAACGCCTGCAAAATC | CCAACGGGTATGAGCTATTCC | Mouse |
| *Grin2b* | TGAACAAAGGCATCAAGCAAATG | CAGTCCAGGGTACGGGTCTT | Mouse |
| *Chat* | CCATTGTGAAGCGGTTTGGG | GCCAGGCGGTTGTTTAGATACA | Mouse |
| *Acox1* | TCGAAGCCAGCGTTACGAG | GGTCTGCGATGCCAAATTCC | Mouse |
| *Acsl4* | CTCACCATTATATTGCTGCCTGT | TCTCTTTGCCATAGCGTTTTTCT | Mouse |
| *Bao* | GGTGGAACCTGGCAACTTCAT | GGTCATCCCACATCTTCTTGG | Mouse |
| *Bcat* | CCCATCGTACCTCTTTCACCC | GGGAGCGTGGGAATACGTG | Mouse |
| *Bdh* | ACAAGACACACGCTGTTGTTT | CTCTTCAAGCTGTCCAGTTCC | Mouse |
| *Bhmt* | TTAGAACGCTTAAATGCCGGAG | GATGAAGCTGACGAACTGCCT | Mouse |
| *Ndor* | AGGCGAATCTCATTAGGGAGC | CAGCAAAATCCATTTGACAGAGG | Mouse |
| *Alox5* | TTGCTCTCACAGTATGACTGGT | AGTATCCACGATCTGCTCGAT | Mouse |
| *Alox12* | TCCCTCAACCTAGTGCGTTTG | GTTGCAGCTCCAGTTTCGC | Mouse |
| *Xdh* | ATGACGAGGACAACGGTAGAT | TCATACTTGGAGATCATCACGGT | Mouse |
| *Acaca* | GATGAACCATCTCCGTTGGC | GACCCAATTATGAATCGGGAGTG | Mouse |

Supplementary Table S3

Primary antibodies of western blot

| Primary antibodies | Cat. No. | Dilution | Manufacturer |
|---|---|---|---|
| GAPDH | AF1186 | 1:2000 | Beyotime Biotechnology Co., Ltd. (Shanghai, China) |
| NLRP3 | AF2155 | 1:1000 | Beyotime Biotechnology Co., Ltd. (Shanghai, China) |
| ASC | AF6234 | 1:1000 | Beyotime Biotechnology Co., Ltd. (Shanghai, China) |
| Caspase-1 | AF1681 | 1:1000 | Beyotime Biotechnology Co., Ltd. (Shanghai, China) |
| GSDMD | HY-P85810 | 1:2000 | MedChemExpress (Monmouth Junction, NJ, USA) |
| ZO-1 | AF5145 | 1:2000 | Affinity Biological Research Center Co., Ltd. (Suzhou, China) |
| Claudin-1 | AF0127 | 1:3000 | Affinity Biological Research Center Co., Ltd (Suzhou, China) |
| Claudin-7 | AF0521 | 1:2000 | Affinity Biological Research Center Co., Ltd (Suzhou, China) |
| Occludin | AF7644 | 1:1000 | Beyotime Biotechnology Co., Ltd. (Shanghai, China) |
| α-SMA | AF1032 | 1:1000 | Affinity Biological Research Center Co., Ltd. (Suzhou, China) |
| Collagen I | AF7001 | 1:1000 | Affinity Biological Research Center Co., Ltd. (Suzhou, China) |
| CTGF | DF-7091 | 1:2000 | Affinity Biological Research Center Co., Ltd. (Suzhou, China) |
| TGF-β1 | K009638P | 1:2000 | Solarbio Life Science Co., Ltd. (Beijing, China) |
| Smad3 | AF1501 | 1:2000 | Beyotime Biotechnology Co., Ltd. (Shanghai, China) |
| Smad4 | AF1291 | 1:2000 | Beyotime Biotechnology Co., Ltd. (Shanghai, China) |
| LC3 | GB115766 | 1:1500 | Servicebio Technology Co., Ltd. (Wuhan, China) |
| Beclin-1 | GB15228 | 1:2000 | Servicebio Technology Co., Ltd. (Wuhan, China) |
| P62 | AG4400 | 1:1000 | Beyotime Biotechnology Co., Ltd. (Shanghai, China) |
| AMPK | AF6423 | 1:2000 | Affinity Biological Research Center Co., Ltd. (Suzhou, China) |
| p-AMPK | AF3423 | 1:2000 | Affinity Biological Research Center Co., Ltd (Suzhou, China) |
| Akt | AF0836 | 1:2000 | Affinity Biological Research Center Co., Ltd (Suzhou, China) |
| p-Akt | AF0016 | 1:2000 | Affinity Biological Research Center Co., Ltd (Suzhou, China) |
| mTOR | AF6308 | 1:2000 | Affinity Biological Research Center Co., Ltd. (Suzhou, China) |
| p-mTOR | AF3308 | 1:2000 | Affinity Biological Research Center Co., Ltd (Suzhou, China) |

## Supplementary Figure S1

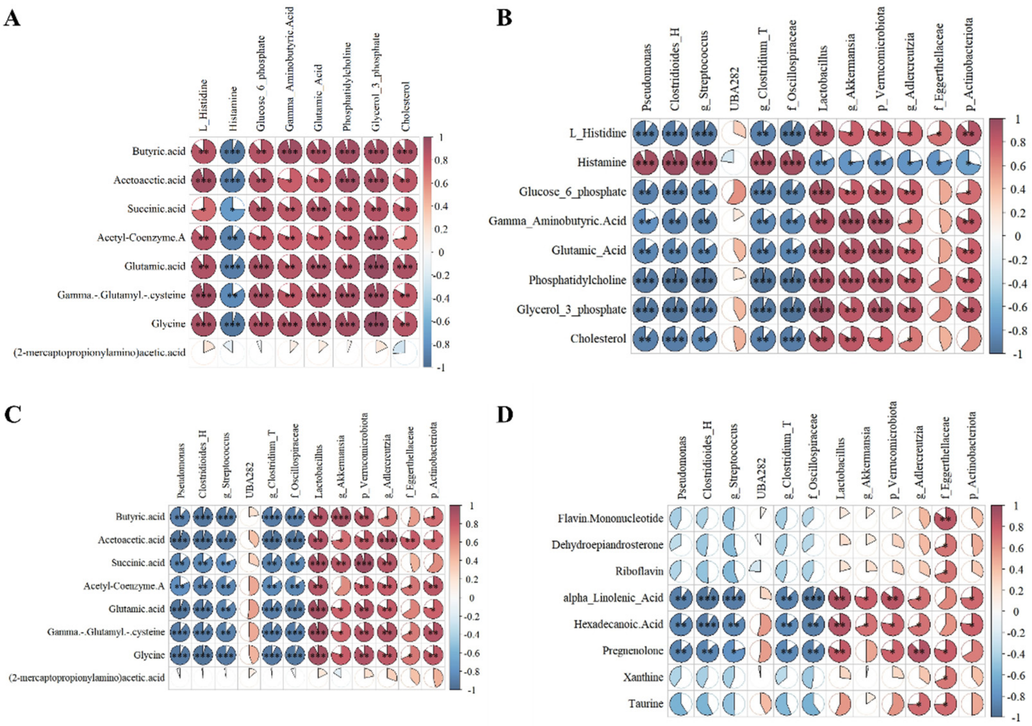


**Figure S1. Correlation Heatmap Analysis of Differential Metabolites and Gut Microbiota Abundance.** (A) Correlation between the relative abundance of differential metabolites in the colon and differential metabolites in the liver. (B) Correlation between the relative abundance of differential metabolites in the liver and the relative abundance of gut microbiota. (C) Correlation between the relative abundance of differential metabolites in the colon and the relative abundance of gut microbiota. (D) Correlation between the relative abundance of differential metabolites in the serum and the relative abundance of gut microbiota. (Red indicates positive correlation, whereas blue indicates negative correlation. Statistical significance is indicated as follows: *$p < 0.05$, **$p < 0.01$, and ***$p < 0.001$).

## Supplementary Figure S2

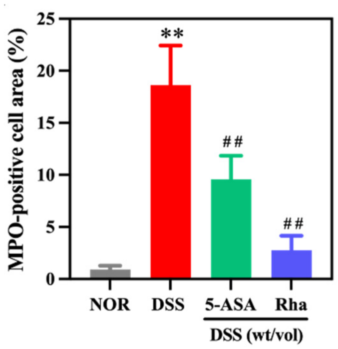


**Figure S2. Immunohistochemical (IHC) semi-quantitative analysis of MPO-positive cells in colon tissue.** MPO-positive cell area (%). Statistical significance is indicated as follows: *$p < 0.05$, **$p < 0.01$ for DSS group vs. NOR group; $^{\#}p < 0.05$, $^{\#\#}p < 0.01$ for treatment groups vs. DSS group.
